# Paperclip as a tool for steatocystoma extraction

**DOI:** 10.1016/j.jdin.2024.07.029

**Published:** 2024-10-26

**Authors:** Nourine A. Kamili, Turkan Banu Karatas, Taha Osman Mohammed, Nujood Alzahrani, Travis W. Blalock

**Affiliations:** Department of Dermatology, Emory University School of Medicine, Atlanta, Georgia

**Keywords:** comedone, cyst, extraction, milium, paperclip, steatocystoma

## Challenge

Steatocystoma multiplex is a benign condition manifesting as papules/nodules on areas dense with pilosebaceous glands (eg, trunk, arms, and face). Although benign, many patients seek treatment. Management options include CO_2_ laser, cryotherapy, isotretinoin, surgical excision, and surgical incision/drainage.^1^ Some of these modalities are expensive, time-consuming, and carry risk of scarring and recurrence.

## Solution

Previously, paperclips have been employed for comedo extraction.^2^ Here, we report the successful extraction of a facial steatocystoma using a paperclip, which served as a simple, inexpensive, and readily available alternative to traditional treatments (Supplementary Video I, available via Mendeley at https://data.mendeley.com/datasets/9d8rtypcjm/1). Additionally, paperclip-assisted extraction may be preferred to many traditional treatments not well-suited for facial lesions prone to scarring.

**Fig 1:**
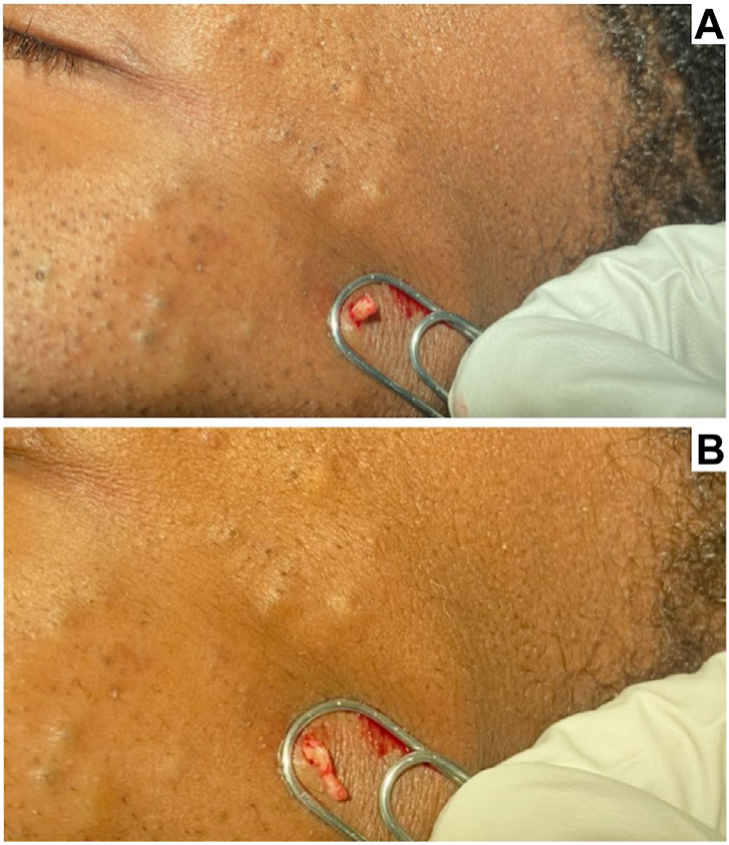
Steatocystoma extraction with paperclip. To extract the steatocystoma, a sterilized paperclip of appropriate size is secured around the lesion (**A**), and a firm, vertical pressure is applied to gently express the cystic contents (**B**).

Paperclips are versatile and can be bent to a diameter slightly larger than the lesion to be extracted,^2^ a feature that is lacking with small comedone extractors. Standard safety procedures should be followed during extraction, such as wearing gloves and prepping skin with antiseptic. The paperclip itself can be sterilized with autoclave or in alcohol. After skin prepping, a small nick into the wall of the cyst can be created with a 15-blade or 18-gauge needle. The paperclip loop is then placed circumferentially around the lesion and firm, vertical pressure is applied to gently express cystic contents through the puncture (Fig 1). Electrosurgical devices can be used for hemostasis.

In conclusion, paperclip-assisted extraction is a versatile and effective option for steatocystoma treatment. This modality is especially advantageous in resource-limited settings, for lesions sensitive to scarring, and/or patients wanting to avoid side effects of traditional treatment options.

## Conflicts of interest

None disclosed.
